# Pediatric cholecystectomy practices and training: an International Multicenter Survey by the European Union of Medical Specialists (UEMS) Section of Paediatric Surgery

**DOI:** 10.1007/s00383-026-06357-y

**Published:** 2026-03-04

**Authors:** Vojtech Dotlacil, Udo Rolle, Lucas Matthyssens, Zane Abola, Kristin Bjørnland, Blanca Capdevila Vilaró, Piotr Czauderna, Mark Davenport, Ede Biro, Niels Bjørn, Julie Galea, Javier Jimenez-Gomez, Stefan Holland-Cunz, Tamas Kovacs, Andriy Kuzyk, Orest Leshnevskyy, Topi Luoto, Dalius Malcius, Carmen Mesas Burgos, Alan Mortell, Oliver J. Muensterer, Matis Märtson, Ivana Sabolić, Tutku Soyer, Konstantinos Velaoras, Milena Senica Verbič, Michal Rygl, Barbora Kucerova

**Affiliations:** 1 Department of Paediatric Surgery, Second Faculty of Medicine, Motol and Homolka University Hospital, Charles University, V Uvalu 84, 150 06 Prague, Czech Republic; 2 Department of Pediatric Surgery and Pediatric Urology, University Hospital Frankfurt, Goethe-University, Frankfurt am Main, Germany; 3 Department of Gastrointestinal and Paediatric Surgery, Princess Elisabeth Children’s Hospital, Ghent University Hospital, Ghent University, Ghent, Belgium; 4 Department of Pediatric Surgery, Clinical University Children’s Hospital, Riga Stradiņš University, Riga, Latvia; 5 Department of Pediatric Surgery, Oslo University Hospital and University of Oslo, Oslo, Norway; 6 Department of Pediatric Surgery, Sant Joan de Déu Barcelona Children’s Hospital, Barcelona, Spain; 7 Department of Surgery and Urology for Children and Adolescents, Medical University of Gdańsk, Gdańsk, Poland; 8 Department of Pediatric Surgery, King’s College Hospital NHS Foundation Trust, London, UK; 9 Division of Paediatric Surgery, Department of Paediatrics, University of Pécs Medical School, Pécs, Hungary; 10 Research Unit for Surgery, Odense University Hospital, Odense, Denmark; 11 Department of Pediatric Surgery, Mater Dei Hospital, Msida, Malta; 12 Department of Pediatric Surgery, Parc Taulí Hospital Universitari, Institut d’Investigació i Innovació Parc Taulí (I3PT-CERCA), Universitat Autònoma de Barcelona, Sabadell, Spain; 13 Department of Pediatric Surgery, University Children’s Hospital Basel (UKBB), Basel, Switzerland; 14 Division of Pediatric Surgery, Department of Pediatrics, University of Szeged, Szeged, Hungary; 15 Department of Pediatric Surgery, Danylo Halytsky Lviv National Medical University, Lviv, Ukraine; 16 Department of Pediatric Surgery, Western Specialized Pediatric Medical Centre, Lviv, Ukraine; 17 Department of Pediatric Surgery, Tampere Center for Child, Adolescent and Maternal Health Research, Faculty of Medicine and Health Technology, Tampere University, Tampere, Finland; 18 Department of Pediatric Surgery, Lithuanian University of Health Sciences, Kaunas, Lithuania; 19 Department of Pediatric Surgery, Karolinska University Hospital, Stockholm, Sweden; 20 Department of Pediatric Surgery, Children’s Health Ireland at Crumlin and Temple Street, Dublin, Ireland; 21 Department of Pediatric Surgery, Dr. von Hauner Children’s Hospital, Ludwig-Maximilians-University Medical Center, Munich, Germany; 22 Department of Pediatric Surgery, Tallinn Children’s Hospital, Tallin, Estonia; 23 Department of Surgery, University Hospital Centre Zagreb, Zagreb, Croatia; 24 Department of Pediatric Surgery, Faculty of Medicine, Hacettepe University, Ankara, Turkey; 25 Department of Pediatric Surgery, Pendeli Children’s Hospital, Athens, Greece; 26 Department of Pediatric Surgery, University Medical Centre Maribor, Maribor, Slovenia

**Keywords:** Pediatric cholecystectomy, Cholelithiasis, Acute calculous cholecystitis, ERCP, Indocyanine green, Surgical training

## Abstract

**Purpose:**

Pediatric cholecystitis and cholelithiasis management is heterogeneous. We surveyed European centers to map current practices, training exposure, and outcomes of pediatric biliary cholecystectomy.

**Methods:**

A 24-item cross-sectional international survey was developed by the European Union of Medical Specialists (UEMS) Section of Paediatric Surgery and distributed to centers in 31 UEMS member states. Items covered institutional resources, indications and timing, surgical approach and adjuncts (ERCP, ICG), training exposure, and center-level outcomes; results are reported as* n* (%), median (IQR). Outcomes were reported at the center level and were self-reported by participating institutions.

**Results:**

Thirty-two centers from 23/31 states responded (74.2%). Pediatric surgeons were primary operators in 84% (shared with adult surgeons in 16%); ERCP access was 66%. Trainee operator share was 22.5% (IQR 5–50) and simulator access 56%. ICG cholangiography was routine in 12.5% and selective in 31%. Acute calculous cholecystitis: 6% always index-admission and 59.4% interval (29–41 days) cholecystectomy; post-ERCP choledocholithiasis: 16% always index-admission cholecystectomy. In 2023, 185 cases were reported: 98.9% laparoscopic with 1.6% conversion; median age 14 years (IQR 12.25–15), operative time 90 min (IQR 60–110), length of stay 2 days (IQR 1–2); 10 complications (5.4%).

**Conclusion:**

Substantial heterogeneity persists in both care pathways and training exposure; most centers lack formal pediatric-specific guidelines, and trainee-led operating remains limited, supporting the need for evidence-based protocols and structured training pathways.

**Supplementary Information:**

The online version contains supplementary material available at 10.1007/s00383-026-06357-y.

## Introduction

While the number of cholecystectomies performed in children is smaller compared to adults, a notable increase has been observed over the past decades [1, 2]. Historically, the primary indication for pediatric cholecystectomy was hematological disease. However, recent trends show a rise in cases linked to alimentary causes, obesity-related gallstone disease, long-term parenteral nutrition, and biliary dyskinesia [3–7]. Unlike in adults, no unified pediatric guidelines exist to inform indications, timing, or perioperative management [8, 9]. Adult recommendations are often extrapolated to children, yet pediatric-specific pathways remain scarce, and real-world practice across Europe is assumed to be heterogeneous. Such variability has implications not only for clinical outcomes but also for training, as exposure to moderately complex procedures—such as cholecystectomy—varies widely between centers.

One goal of the European Union of Medical Specialists (Union Européenne des Médecins Spécialistes—UEMS) is to harmonize specialist practice and training across Europe, and the UEMS Section of Paediatric Surgery is dedicated to this mission. Yet current data describing pediatric surgical practice among member countries remain limited. Cholecystectomy represents a moderately complex procedure and may therefore serve as an index operation to assess current practice and training patterns for moderately complex cases.

To assess and characterize current European practices, training exposure, and outcomes, we conducted a standardized UEMS-endorsed survey among member countries in an effort to support the development of pediatric surgical standards and recommendations.

## Methods

### Study design and setting

An international, cross‑sectional survey of pediatric surgical centers (varying institutional profiles) affiliated with the UEMS Section of Paediatric Surgery. The instrument comprised 24 items across two sections: (1) Institutional practices and training; (2) Center‑level outcomes. Prior to dissemination, the questionnaire was piloted with the Executive Board of the UEMS Section of Paediatric Surgery to ensure clarity and content validity. The full survey instrument is available in Supplementary Appendix 1.

### Participants and eligibility

Centers performing cholecystectomy for symptomatic biliary disease in patients ≤ 18 years were included.

Exclusion criteria were: non-calculous indications, hemolytic or other hematological disorders, and prophylactic or incidental cholecystectomy performed in conjunction with splenectomy or major hepatobiliary reconstruction (distinct biology and indications).

### Survey administration

The survey was distributed electronically (SurveyMonkey^®^, San Mateo, CA, USA) to delegates of 31 UEMS member states. Responses were anonymized at the center level. Participation implied consent. No patient‑identifiable data were collected. We allowed multiple participating centers per country. Each response represented one center.

### Variables and outcomes

Items captured: surgeon specialty (pediatric/adult); access to Endoscopic Retrograde Cholangiopancreatography (ERCP); use of Indocyanine Green (ICG) fluorescence; trainee participation (perform/assist); simulator access; drain and antibiotic policies; timing strategies for acute calculous cholecystitis (ACC) and for choledocholithiasis treated with ERCP (conditional, non-mutually exclusive responses). Centers reporting 2023 outcomes provided numbers of cholecystectomies (laparoscopic/open and conversions), median (IQR) age, operative duration, length of stay (LOS), and postoperative complication grades (Clavien–Dindo classification).

### Statistical analysis

Categorical variables are reported as* n* (%). Continuous variables are summarized as median (IQR). Group comparisons were performed using the Mann–Whitney U test for continuous variables and Fisher’s exact test for categorical variables. Denominators vary by item because not all centers provided 2023 outcome data; item-specific denominators are reported throughout. All tests were two-sided; *P* < 0.05 was considered statistically significant. Analyses were conducted in GraphPad Prism 8.1.1 (GraphPad Software, San Diego, CA, USA).

### Ethics

This study surveyed anonymized institutional practices and center‑level outcomes without patient identifiers. In line with participating centers’ policies and national regulations, formal ethics committee approval was not required for this health‑services survey.

## Results

### Centers and platforms

**Fig. 1:**
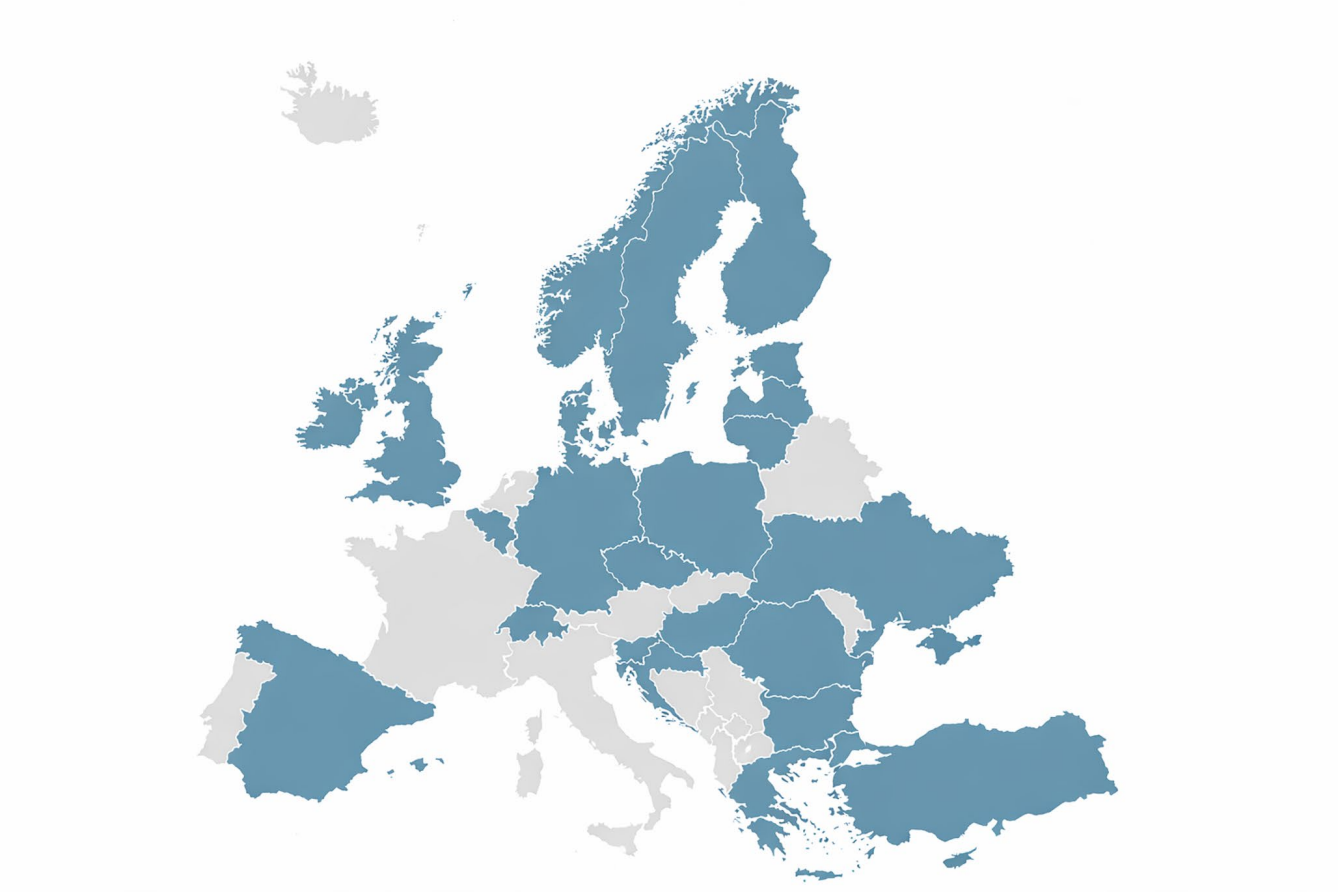
Geographic distribution of participating centers across Europe. Highlighted states indicate at least one responding center. The number of responding centers per state is provided in Supplementary Appendix 2

Thirty‑two centers from 23 (74.2%) UEMS member states (Fig. 1) responded to the practices section; 19/32 (59%) also provided 2023 outcome data. Most centers reported that pediatric surgeons are doing cholecystectomy (27; 84%), while 5 (16%) report shared practice with adult surgeons.

ERCP access for children was available in 21 (66%) centers. ICG fluorescence cholangiography was used in all cases in 4 (13%), selectively in 10 (31%), and not used in 18 (56%) centers.

Laparoscopic simulator access for trainees was available in 18 centers (56%). Trainees were permitted to perform cholecystectomies in 24 centers (75%; median operative share 22.5%, IQR 6–50), and they assisted in 30 centers (94%; median participation 70%, IQR 30–100).

### Timing strategies

**Cholecystectomy for acute calculous cholecystitis (ACC) -** Two of 32 centers (6%) always perform cholecystectomy during the index admission; 24 (75%) decide based on disease severity/response; 6 (19%) never perform index‑admission surgery. After conservative therapy, delayed cholecystectomy is an established indication in 19 (59%) centers. The most common planned interval from discharge to surgery was 29–41 days (12; 38%). Given the small number of events, no formal comparative statistics were performed.

**Cholecystectomy after ERCP for choledocholithiasis -** Five of 32 centers (16%) always perform the index-admission cholecystectomy; 16 (50%) decide based on the course and local logistics; 11 (34%) never perform the index‑admission surgery.

### Perioperative policies

Routine drain placement during planned laparoscopic cholecystectomy was performed routinely in only 1 (3%) center, used selectively based on intraoperative findings in 8 (25%) centers, applied according to surgeon preference in 3 (9%) centers, and was never used in 20 (63%) centers. Perioperative antibiotics were administered prophylactically (single pre-incision dose) in 24 (75%) centers; 5 (16%) centers never administered antibiotics, and 3 (9%) centers used multi-dose therapeutic regimens.

### Adherence to guidelines

Most centers (23; 72%) reported following no specific recommendations. Five (16%) referenced adult guidelines, three (9%) local institutional protocols, and one (3%) a pediatric‑specific guideline.

### Operative outcomes in 2023 (center‑level)

Across responding centers, 185 pediatric cholecystectomies were performed in 2023; 183 (98.9%) were laparoscopic with three (1.6%) conversions. Median age at surgery was 14 years (IQR 12.25–15). Median operative duration was 90 min (IQR 60–110). Median postoperative LOS was 2 days (IQR 1–2). No center performed more than 20 cholecystectomies annually, whereas 30% performed less than five cases per year (Table 1). Ten complications were reported (5.4%). Postoperative complications, reported by Clavien–Dindo grade only, are summarized in Table 2. Exploratory analyses did not identify a statistically supported association between annual center volume and postoperative outcomes (*p* = 0.30); however, the low event rate limited statistical power for reliable volume–outcome inference.

**Table 1:** Annual case volume per center (2023)

Annual case volume	Number of centers	Percentage
≤ 5 procedures	6	30%
6–10 procedures	6	30%
11–20 procedures	8	40%
> 20 procedures	0	0%

Case-volume data were available for 20 centers; one center reported volume only, so complete 2023 outcomes were available for 19

**Table 2:** Postoperative complications in 2023 by Clavien–Dindo classification

Clavien–Dindo classification	*n*	% of all cases	% of complications
I	6	3.2%	60.0%
II	3	1.6%	30.0%
III	1	0.5%	10.0%
IV	0	0.0%	0.0%
V	0	0.0%	0.0%
Total	10	5.4%	100.0%

Percentages are calculated using the total number of pediatric cholecystectomies (*N* = 185) as the denominator for the “% of all cases” column

## Discussion

This UEMS-led survey addresses an area with limited coordinated data by providing a pan-European overview of pediatric cholecystectomy practices. It demonstrates pronounced variation in timing strategies, use of adjuncts such as ERCP and ICG, and training exposure. Although laparoscopy has been adopted almost universally and conversion and complication rates are low, no formal, widely endorsed pediatric-specific guidelines exist. Centers instead extrapolate adult recommendations or rely on local protocols. These observations mirror recent national and international snapshots showing heterogeneity in pediatric surgical pathways.

### Timing of cholecystectomy after ACC and ERCP

Adult recommendations favor early cholecystectomy for ACC and index‑admission or early cholecystectomy after ERCP for choledocholithiasis/mild biliary pancreatitis to reduce interval events and readmissions [9, 10]. Some database studies now point in the same direction, that index‑admission cholecystectomy for pediatric ACC is associated with substantially lower 30‑day readmissions compared to delayed surgery, and for pancreatitis, index‑admission cholecystectomy lowered readmission odds by ~ 84% [11, 12]. Earlier multicenter pediatric series for gallstone pancreatitis similarly showed lower recurrence with index-admission surgery compared with delayed approaches [13]. However, caution remains appropriate in severe biliary pancreatitis, where adult data suggest deferring cholecystectomy in moderate-to-severe disease [14]. Overall, accumulating pediatric data—combined with the robust evidence base in adults for mild disease—supports harmonizing towards earlier definitive surgery where resources and expertise allow, while deferring in more severe pancreatitis. While external evidence increasingly supports earlier surgery in mild disease, our survey describes practice variation. As a descriptive survey, our data do not allow outcome-based comparisons between timing strategies; therefore, recommendations rely on existing literature and require prospective pediatric validation, reinforcing the need for pediatric-specific guidelines.

### Laparoscopy and ICG

In our cohort, a laparoscopic approach was chosen in nearly all cases (98.9%), with only 1.6% conversion to open surgery despite the heterogeneity of centers and relatively low annual volumes. These numbers align with the literature showing that laparoscopic cholecystectomy (LC) is the preferred method for pediatric gallstone disease owing to feasibility, low conversion risk, and low perioperative morbidity [15]. Indocyanine green fluorescence cholangiography has gained prominence for enhancing biliary anatomy visualization without radiation and may shorten key operative steps; pediatric series and comparative studies support its safety and utility [16–18]. Among participating centers, ICG was available in nearly 70%, but used routinely in only 12.5%, with most centers applying it selectively. Potential barriers to broader adoption may include equipment availability and cost, as well as variability in institutional workflows for dye administration, uncertainty regarding optimal dosing and timing, and the learning curve associated with fluorescence interpretation. Given this pattern, pediatric-specific guidance on indications, dosing, and timing may help standardize its adoption.

### Role of ERCP and peri‑diagnostic risk stratification

Two‑stage management with ERCP followed by cholecystectomy is common across respondents in patients with choledocholithiasis, but access to pediatric ERCP remains uneven. A 2023 meta‑analysis (*n* > 5,000 pediatric ERCPs) reported ~ 95% procedural success and ~ 7% overall adverse events, including ~ 4% post‑ERCP pancreatitis, comparable to adult series [19]. Where advanced endoscopy is limited, robust pre‑test stratification helps avoid non‑therapeutic ERCP. The pediatric DUCT score (CBD ≥ 6 mm, ultrasound‑proven duct stone, total bilirubin ≥ 1.8 mg/dL) offers high specificity and practical risk tiers for choledocholithiasis, enabling targeted ERCP or direct cholecystectomy with on‑table imaging where appropriate [20]. Although we did not quantify the use of formal risk stratification tools, ERCP availability (~ 66% of centers) appeared to influence timing behavior: Centers with ERCP access were more likely to complete the index-admission cholecystectomy after duct clearance (24% vs. 0%).

### Pediatric surgeons in training and alignment with European training requirements

Despite laparoscopic cholecystectomy being the preferred approach, the actual proportion of cases performed by trainees in pediatric surgery remained low across centers (median 22.5%, IQR 5–50), likely reflecting a combination of low annual case volumes and variability in opportunities for supervised operative training. This mirrors European reports that young pediatric surgeons have limited LC exposure within MIS curricula and would benefit from structured simulation [21–23], with training in higher-volume centers representing one possible complementary approach [24–26].

In adult surgical training, multiple studies suggest that 30–60 laparoscopic cholecystectomies are typically required to achieve procedural competency, with some trainees needing even more cases to reach proficiency. For example, a large Japanese study observed that operative time among trainees stabilized after approximately 60 cases [27], and a systematic review reported a broad range for competency acquisition, with thresholds spanning from 13 to over 90 procedures depending on assessment criteria [28].

In contrast, pediatric case volumes are inherently lower, and as reflected in our survey, trainee participation remains limited. Considering that many responding centers performed fewer than 20 cholecystectomies annually, it is unlikely that pediatric surgery trainees could achieve exposure comparable to adult benchmarks. Learning-curve data for pediatric laparoscopic cholecystectomy are currently lacking, further emphasizing the need to define realistic training targets in this context.

Within the UEMS European Training Requirements framework, pediatric laparoscopic cholecystectomy could represent one of the ideal index minimally invasive procedures, for which centers might specify: (i) suggested minimum case numbers as first surgeon/assistant; (ii) routine documentation of the Critical View of Safety; (iii) demonstrated competency with bailout strategies; and (iv) ensured access to simulation [29]. Our findings support increasing supervised trainee participation and suggest formalizing access to simulation to address the current gap.

### Strengths and limitations

Strengths of this study include UEMS endorsement, international multicentric broad geographic coverage, and linkage of practice patterns with recent outcomes. Limitations include self-reported, center-level data, incomplete national coverage, institutional self-selection, descriptive analysis without patient-level risk adjustment, and retrospectively reported complications, which may underestimate true postoperative event rates. Additional limitations are inherent to the survey-based design.

## Conclusions

The results of our study reveal considerable variability in the management of pediatric cholecystectomy across Europe, likely reflecting the absence of evidence-based guidelines. While pediatric laparoscopic cholecystectomy is almost universally adopted, practices differ notably in timing strategies, access to ERCP, and perioperative policies. Trainee involvement as primary surgeon was low across centers, highlighting the need to strengthen structured training opportunities. These findings underscore the need for standardized, pediatric-tailored recommendations to support consistent and high-quality care across Europe.

## Supplementary Information

Below is the link to the electronic supplementary material.

Supplementary Material 1

Supplementary Material 2

## Data Availability

the anonymized survey instrument and aggregated dataset are available as Offline Resources and upon reasonable request to the corresponding author.
