# Supplementary material for: Pediatric cholecystectomy practices and training: an International Multicenter Survey by the European Union of Medical Specialists (UEMS) Section of Paediatric Surgery
Source: Pediatr Surg Int. 2026 Mar 4;42(1):134. doi: 10.1007/s00383-026-06357-y (PMC12960363; doi:10.1007/s00383-026-06357-y)
Supplement: Supplementary file 1 — Supplementary Material 1 [file 383_2026_6357_MOESM1_ESM.pdf]

Cholecystectomy in children due to biliary etiology

General part of the questionnaire

It includes general questions on care management and questions about doctors in training.

* 1. Respondent information

Full Name

Institution

Country

Email Address

* 2. Cholecystectomy in children at your institution is performed by?

- ☐ Pediatric surgeon
- ☐ Adult surgeon
- ☐ Both
- ☐ None, the patient is referred to a higher pediatric center

* 3. Does your institution have the capability to perform ERCP (endoscopic retrograde cholangiopancreatography) in children?

- ◯ Yes
- ◯ No

* 4. Do pediatric surgeons in training perform cholecystectomies at your institution? If yes, what is their share of the total number (percentage)

Yes/ No

If yes, what was their share of the total in 2023 (in percent)?

* 5. Do pediatric surgeons in training assist cholecystectomies at your institution? If yes, what is their share of the total number (percentage)?

Yes/ No

If yes, what was their share of the total in 2023 (in percent)?

* 6. Do pediatric surgeons in training at your institution have access to laparoscopic simulators for cholecystectomy?

- ◯ Yes
- ◯ No

* 7. Do you use indocyanine green (ICG) fluorescence for cholecystectomy at your institution?

- ◯ Yes, in all cases
- ◯ Depending on the surgeon’s decision
- ◯ No
- ◯ Other (please specify):

* 8. For patients acutely admitted with calculous cholecystitis, is cholecystectomy performed during the same hospitalization (acute cholecystectomy)?

- ◯ Yes, always
- ◯ It depends on the duration of the symptoms
- ◯ Depending on the surgeon’s decision
- ◯ No, never
- ◯ Other (please specify)

* 9. If yes, within how many days after the onset of symptoms do you perform cholecystectomy?

- ◯ 3
- ◯ 5
- ◯ 7
- ◯ Other (please specify)

* 10. After conservative treatment of acute calculous cholecystitis, is there an indication for delayed cholecystectomy at your institution?

- ◯ Yes, always
- ◯ Depending on the surgeon’s decision
- ◯ No, never
- ◯ Other (please specify)

* 11. If yes, within what time interval from the resolution of inflammation is the planned cholecystectomy performed (count from the date of discharge)?

- ◯ 15 - 28 days
- ◯ 29 - 41 days
- ◯ 42 or more days
- ◯ Other (please specify)

* 12. After ERCP for choledocholithiasis, do you perform cholecystectomy during the same hospitalization?

- Yes, always
- Depending on the surgeon’s decision
- No, never
- Other (please specify)

* 13. Do you place a drain during planned laparoscopic cholecystectomy?

- Yes, always
- Depending on intraoperative findings
- Depending on the surgeon’s decision
- No, never

* 14. Are antibiotics administered for planned laparoscopic cholecystectomy?

- No, never
- Prophylactically (1 dose at the beginning of the operation)
- Therapeutically (more than 1 dose)

Other (e.g., according to the surgeon, 3 doses, 1 day, etc.):

15. What surgical guidelines do you follow for this topic, if any (Please specify (+ DOI if applicable):

Cholecystectomy in children due to biliary etiology

A specialized section of the questionnaire

It includes questions on specific procedures and the number of patients in your department in 2023.

* 16. How many cholecystectomies due to biliary etiology were performed at your institution in 2023 (all together)?

Primarily laparoscopically

Number of conversions

Primarily open

17. How many women were in the cohort?

0 150

18. Age (years) at the time of planned cholecystectomy (median and interquartile range)?

Median:

Q1:

Q3:

19. Duration (minutes) of the surgery for planned cholecystectomy (median and interquartile range)?

Median:

Q1:

Q3:

20. The length of hospital stay (days) after planned cholecystectomy (median and interquartile range)?

Median:

Q1:

Q3:

* 21. How many cases of acute calculous cholecystitis were treated at your hospital in 2023?

0 200

* 22. How many cholecystectomies were performed acutely (during the same hospitalization) for acute cholecystitis at your institution in 2023?

Primarily laparoscopically:

Number of conversions (if primarily laparoscopically):

Primarily open:

* 23. How many conservatively treated patients with acute cholecystitis, initially indicated for delayed cholecystectomy, had to undergo emergency surgery in 2023?

Number of planned delayed surgeries:

Number of emergency surgeries:

24. Number of patients with complications according to Clavien-Dindo classification after planned cholecystectomy (number)?

C-D I:	
C-D II:	
C-D III:	
C-D IV:	
C-D V:	
