# Supplementary material for: Pediatric cholecystectomy practices and training: an International Multicenter Survey by the European Union of Medical Specialists (UEMS) Section of Paediatric Surgery
Source: Pediatr Surg Int. 2026 Mar 4;42(1):134. doi: 10.1007/s00383-026-06357-y (PMC12960363; doi:10.1007/s00383-026-06357-y)
Supplement: Supplementary file 2 — Supplementary Material 2 [file 383_2026_6357_MOESM2_ESM.pdf]

**Country**	**Number of centers**
Belgium	1
Croatia	1
Czech Republic	1
Denmark	2
Estonia	1
Finland	1
Germany	2
Greece	2
Hungary	2
Ireland	1
Latvia	1
Lithuania	1
Malta	1
Norway	1
Poland	1
Romania	1
Slovenia	1
Spain	5
Sweden	1
Switzerland	1
Türkiye	1
Ukraine	2
United Kingdom	1

Note: 32 responding centers across 23 countries.
